# The association between the angiotensin-converting enzyme-2 gene and blood pressure in a cohort study of adolescents

**DOI:** 10.1186/1471-2350-14-117

**Published:** 2013-11-05

**Authors:** Lucile Malard, Lisa Kakinami, Jennifer O’Loughlin, Marie-Hélène Roy-Gagnon, Aurélie Labbe, Louise Pilote, Pavel Hamet, Johanne Tremblay, Gilles Paradis

**Affiliations:** 1 Department of Epidemiology, Biostatistics and Occupational Health, McGill University, Montréal, QC, Canada; 2 Institut de Santé Publique d’Epidémiologie et de Développement (ISPED), Université Bordeaux Segalen, Bordeaux, France; 3 CRCHUM, Département de médecine sociale et préventive, Université de Montréal, Montréal, QC, Canada; 4 Sainte-Justine University Hospital Research Center, Université de Montréal, Montréal, QC, Canada; 5 Department of Epidemiology and Community Medicine, University of Ottawa, Ottawa, ON, Canada; 6 Department of Medicine, McGill University and McGill University Health Center, Montréal, QC, Canada; 7 Department of Medicine, Centre de recherche du CHUM, Université de Montréal, Montréal, QC, Canada

**Keywords:** Blood pressure, Angiotensin-Converting Enzyme-2, Adolescent, Cohort study

## Abstract

**Background:**

The Angiotensin-Converting Enzyme-2 (ACE2) gene, located on chromosome X, is believed to be implicated in blood pressure regulation. However the few studies that have examined this association have yielded mixed results. The objective of this study was to assess the association between tag single nucleotide polymorphisms (SNPs) in the angiotensin-converting enzyme-2 gene with blood pressure and blood pressure change in adolescents.

**Methods:**

Participants in the Nicotine Dependence in Teens (NDIT) cohort study with blood or saliva samples and at least 3 blood pressure measurements over 5 years were included in the analytic sample (n = 555). Linear growth curve models stratified on sex and ethnicity were used to assess the association between four tag SNPs in the ACE2 gene and systolic (SBP) and diastolic blood pressure (DBP), and blood pressure change.

**Results:**

In males of European descent, rs2074192 and rs233575 were significantly associated with SBP and DBP, and rs2158083 was associated with SBP. In French Canadian males, rs233575 and rs2158083 were significantly associated with DBP. Among females of European descent, rs2074192, rs233575, and rs2158083 were significantly associated with change in SBP over 5 years.

**Conclusions:**

This is the first study to assess the association between the ACE2 gene with blood pressure and blood pressure change in a cohort of adolescents. Results indicate that several ACE2 gene SNPs are associated with blood pressure or blood pressure change in persons of European descent. However the therapeutic potential of these SNPs should be explored.

## Background

Hypertension (HTN) is a major risk factor for cardiovascular disease. Half of all strokes and ischemic heart disease, and 25% of other cardiovascular diseases are due to elevated blood pressure [1]. In 2000, the prevalence of HTN as defined by systolic blood pressure (SBP) ≥ 140 mmHg or diastolic blood pressure (DBP) ≥ 90 mmHg was 26% worldwide [2]. It is estimated that the prevalence will reach 29% by 2025 [2]. Genetic influences on blood pressure have been clearly established, with heritability estimates between 0.20 and 0.60 [3-6]. It is thus important to better understand which genes are implicated in the regulation of blood pressure and how these genes influence blood pressure levels. Increased understanding of genetic risk could help identify new therapeutic targets for HTN, as well as distinguish population subgroups that may benefit from closer monitoring and more intensive prevention efforts.

The Angiotensin-Converting Enzyme-2 (ACE2) is a negative regulator of the renin-angiotensin aldosterone system [7-9] and plays an important role in blood pressure homeostasis [9]. ACE2, a homolog to the Angiotensin-Converting Enzyme (ACE), contains a single catalytic site that is 42% similar to the ACE catalytic sites, and ACE2 and ACE are believed to have antagonistic effects on blood pressure [7,8,10,11]. While animal models demonstrate an increase in Ang II peptide levels (a vasoconstrictor substance) with ACE2 inhibition [9,12], studies of the association between blood pressure and ACE2 single nucleotide polymorphisms (SNPs) have yielded mixed results in humans. Studies in Australian, German, and Han Chinese populations did not find any association between ACE2 SNPs and blood pressure [11,13,14], while studies in Chinese populations that were not Han Chinese found statistically significant associations between two ACE2 SNPs and blood pressure [15,16].

Past studies on ACE2 SNPs were conducted in adults and were primarily case–control or cross-sectional. The results are subject to confounding by preexisting chronic diseases or treatments for high blood pressure. Because young people generally do not have chronic disease or take blood pressure medications, assessing the association between blood pressure and ACE2 using data from adolescents may improve our understanding of the implication of this gene in blood pressure regulation. Thus, the aim of this study was to assess the association between blood pressure and ACE2 gene SNPs in an ongoing cohort study of healthy adolescents.

## Methods

### Study population

The Nicotine Dependence in Teens (NDIT) Study is an ongoing prospective cohort investigation designed to assess the development of nicotine dependence symptoms after cigarette smoking onset in adolescents [17]. Participants were recruited in a convenience sample of ten French and English schools in Montreal. Schools were located in urban, suburban and rural settings, and participants were of varying socio-economic status. A total of 1,293 students in grade 7 (12–13 years of age) participated in the baseline assessment in 1999–2000.

Participants completed 45-minute self-reported questionnaires every 3–4 months during the 10-month school year, for a total of 20 waves of data collection during high school. Data from the baseline and the first five years of follow-up were used in this study. Blood pressure and anthropometric measures were obtained in survey cycle 1, 12 and 19 during secondary school (when participants were 12.7, 15.1, and 17.0 years of age on average, respectively). The blood or saliva samples for DNA extraction were provided by 852 of the 1293 participants (65.9%) in 2002 and 2007. All participants and a parent or guardian provided written informed consent at baseline; a separate consent was provided by parents for the blood draw in grade 9 (when participants were 14–15 years of age), and informed consent was obtained from participants (who had attained legal age) for saliva samples in 2007. The study was approved by Institutional Review Boards at the CRCHUM, the Direction de santé publique de Montreal and McGill University.

### Measurements

After voiding and a 5-minute rest, blood pressure was measured on the right arm of seated subjects according to a standardized protocol by a trained and certified technician. Measurements were made with an automated oscillometric device (Dinamap XL, model CR9340; Critikon Company LLC, Tampa, Florida) calibrated against a mercury sphygmomanometer before each data collection period. SBP and DBP were measured 3 times at 1-minute intervals. If the SBP and DBP measurements differed by more than 10 and 5 mmHg respectively, fourth and fifth measurements were obtained. The mean of the two closest measurements (after exclusion of the first measurement) was used for analysis.

### Covariates

Duplicate measurements of height and weight were obtained with participants wearing light clothing without shoes, using a stadiometer (model 214 Road Rod; Seca Corp., Hanover, MD, USA) for height and a scale (floor model 761; Seca Corp., Hanover, MD, USA) for weight. A third measurement was obtained if the difference between the two measurements was greater than 0.5 cm for height and 0.2 kg for weight. The average of the two closest measurements was used in the analysis. To assess inter-rater reliability of height and weight, measures of every 1 in 10 participants were repeated. Inter-rater reliabilities were 0.99 for height and weight [18,19]. Sex- and age-specific body mass index (BMI) percentiles were assessed and youth were categorized as overweight or obese if the BMI was ≥ 85th percentile according to the Centers for Disease Control and Prevention guidelines [20]. The date of birth and sex of participants were self-reported in the questionnaire.

### SNP selection and genotyping

We used the greedy pairwise tagging approach [21] as implemented in the software Haploview [22] in order to tag polymorphisms present in the HapMap CEU data. We selected SNPs with a minor allele frequency >5%. We used a linkage disequilibrium (LD) cutoff of r2 > 0.8, ensuring that each unmeasured SNP is in strong LD with a tag SNP. We tagged polymorphisms from 10 kb before to 10kb after the location of ACE2 using genomic positions according to NCBI build 36. The four selected tag SNPs (rs2074192, rs233575, rs2158083 and rs1978124) were genotyped at the McGill University and Genome Quebec Innovation Center using iPLEX Gold technology (Sequenom, San Diego, Calif, USA).

### Data analysis

Participants with only one or two blood pressure measurements during the five years of high school were excluded from analyses because of the potential for over fitting. Because females begin puberty before males, and the evolution of blood pressure differs between males and females in adolescence [23,24], the analyses were stratified by sex. Differences in demographic and baseline characteristics between males and females were tested with chi-squares and t-tests, and differences in SBP or DBP change over time were tested with general linear models and paired t-tests as appropriate. Because the ACE2 gene is located on the X chromosome, males have only one allele of the ACE2 gene while females have two. However, which of the alleles is expressed is unknown in heterozygous females, and therefore we restricted our analyses to males and homozygous females for each specific SNP, as has been done in the literature [25]. We tested Hardy-Weinberg Equilibrium in females. Differences in allele frequencies between males and females and LD were assessed with χ^2^ tests.

Due to evidence of heterogeneity between ethnicities, analyses were also stratified by ethnicity (French Canadian, European, Other). Analyses assessing the association between baseline blood pressure and the major and minor SNP genotypes were conducted with linear regression analyses. Major and minor SNP genotypes were identified in accordance with the dbSNP database [26]. A linear growth curve model with an unstructured error covariance structure was used to assess the association between SBP or DBP, and *change* of SBP or DBP with the four ACE2 SNPs. SBP and DBP were modeled as continuous variables in separate models; time was represented by age centered at 12 years, ethnicity was defined as French Canadian, European descent, or Other (biological parents from Asian, Spanish, African, etc., countries), and the major allele genotypes served as the reference category (GG for rs2074192 and rs1978124, and TT for rs233575 and rs2158083 for females; G for rs2074192 and rs1978124, and T for rs233575 and rs2158083 for males). All models adjusted for height and whether or not the participant was overweight or obese. Statistical analyses were carried out using SAS 9.2 software (SAS Institute Inc., Cary, NC, USA).

## Results

Participants with fewer than three blood pressure measurements (n = 570), or those who did not provide a blood or saliva sample (n = 168) were excluded. Therefore a total of 555 participants of the original 1,293 were retained for analyses. At baseline, adolescents who were excluded were significantly younger than those retained in analyses (12.6 years old compared to 12.9, *p* < 0.05) but did not differ in sex, ethnicity, whether or not they were overweight or obese, SBP or DBP (*p* > 0.05, data not shown).

Over half (52%) of the participants in the complete sample (n = 555) were male (Table 1). Females were not significantly different from males in age, ethnicity, BMI, or baseline SBP or DBP. Mean SBP at baseline was 105 mmHg and 104 mmHg in males and females, respectively and mean baseline DBP was 56 mmHg and 56 mmHg in males and females respectively. SBP was moderately correlated with DBP in wave 1, 12, and 19 (Pearson r = 0.55, 0.59, and 0.59, respectively). These results were unchanged after excluding heterozygote females (data not shown). SBP was statistically significantly lower in females than males at both follow-ups (*p* < 0.0001 in wave 12 and wave 19), but DBP was not statistically significantly different (*p* > 0.05 in wave 12 and wave 19). SBP increased 10.1 mmHg on average over the 5-year follow-up (*p* < 0.0001) in males, and increased 2.0 mmHg on average in females (*p* = 0.0003), a statistically significant difference (*p* < 0.0001). DBP increased 3.4 mmHg on average over the 5-year follow-up in males (*p* < 0.0001), and 2.2 mmHg on average in females (*p* < 0.0001), but the difference was not statistically significant by sex (*p* > 0.05). These results were unchanged after excluding heterozygote females (data not shown).

**Table 1:** Selected characteristics (n = 555) of the NDIT participants, 1999-2005

**Characteristic**^**1**^	**Male (n = 265)**	**Female (n = 290)**	***p***^***2***^
Age, years, mean (SD)^3^	12.7 (0.42)	12.6 (0.39)	0.06
Ethnicity, %			
French Canadian	29	32	0.71
European descent	47	43	
Other	24	25	
Overweight or obese, %	27	21	0.11
SBP, mmHg, mean (SD)			
Baseline (~13 years of age)	105.3 (10.2)	104.5 (9.7)	0.36
Wave 12 (~15 years of age)	109.1 (11.2)	104.1 (10.0)	<0.0001
Wave 19 (~17 years of age)	115.4 (11.5)	106.6 (10.4)	<0.0001
DBP, mmHg, mean (SD)			
Baseline (~13 years of age)	55.6 (6.0)	56.5 (6.2)	0.08
Wave 12 (~15 years of age)	56.7 (5.7)	57.4 (6.5)	0.19
Wave 19 (~17 years of age)	58.9 (6.3)	58.6 (6.3)	0.58

^1^Baseline measurements unless otherwise noted; ^2^difference between males and females; ^3^SD: Standard deviation.

As mentioned in our methods, we excluded heterozygous females from analyses for each specific SNP resulting in analytic samples that ranged from 407–415 participants. Allele frequencies stratified by ethnicity were compared between males and females and were not statistically significantly different (Table 2), except for rs2074192 which had an A allele frequency of 25% in French Canadian males compared to 44% in French Canadian females (*p* = 0.01), and an A allele frequency of 27% in males of other ethnicity compared to 41% in females of other ethnicity (*p* = 0.01). SNPs rs233575, rs2158083, and rs1978124 were in Hardy-Weinberg Equilibrium (*p* > 0.05, data not shown). All SNPs were in linkage disequilibrium with each other (Additional file 1: Table S1).

**Table 2:** Frequency of the minor alleles stratified by sex and ethnicity (NDIT Study, 1999–2005)

	**French Canadian**			**European**			**Other**		
**SNP (Minor/Major)**^**1,2**^	**Male**	**Female**	***p***^**3**^	**Male**	**Female**	***p***^**3**^	**Male**	**Female**	***p***^**3**^
rs2074192 (A/G)	24.7%	43.9%	<0.01	47.1%	46.8%	0.93	26.6%	40.7%	0.01
rs233575 (C/T)	41.9%	36.8%	0.35	37.5%	39.9%	0.58	33.3%	23.2%	0.07
rs2158083 (C/T)	37.7%	37.5%	0.98	40.3%	40.4%	0.99	33.3%	23.9%	0.09
rs1978124 (A/G)	45.4%	49.4%	0.46	55.4%	52.0%	0.46	31.2%	31.0%	0.96

^1^Depending on the SNP, *n* ranged from 142 to 150 female, and 257 to 264 male participants; ^2^Categorizations of major and minor genotypes in accordance with dbSNP database; ^3^Comparison of frequencies between males and females.

### SNPs and *baseline* blood pressure

In multivariable linear regression analyses, SNP rs1978124 was not associated with SBP or DBP at baseline in either males or females (*p* > 0.05 for all, Additional file 2: Table S2). Among males of European descent, carrying the G allele of SNP rs2074192 was associated with higher DBP at baseline (56 mmHg vs 53 mmHg, *p* < 0.05), and the T allele of SNP rs233575 was associated with lower SBP (104 mmHg vs 108 mmHg, *p* < 0.05) and DBP (54 mmHg vs 56 mmHg, *p* < 0.05). French Canadian females carrying the T allele for rs233575 or rs2158083 had baseline DBP that was 4 mmHg higher (59 mmHg vs 55 mmHg) than French Canadian females carrying the C allele (*p* < 0.05).

### SNPs and blood pressure, or SNPS and blood pressure *change* (males)

Sex- and ethnicity-stratified analyses suggested that after adjusting for height and weight status, SBP and DBP in males of European descent carrying the A allele of rs2074192 were 3.3 mmHg lower (*p* = 0.02) and 2.3 mmHg lower (*p* = 0.003), respectively than in males of European descent carrying the G allele (Table 3). In contrast, the SBP and DBP of males of European descent carrying the rs233575 C allele were 3.6 mmHg (*p* = 0.01) and 2.4 mmHg higher (*p* = 0.003), respectively, than in males of European descent carrying the T allele. In addition, males carrying the C allele of rs2158083 had SBP that was 3.4 mmHg higher (*p* = 0.02) than males carrying the T allele. French-Canadian males with the C allele of rs233575 and rs2158083 had 2.9 mmHg (*p* = 0.001) and 2.7 mmHg (*p* = 0.01) lower DBP than males with the T allele of these respective SNPs. There were no statistically significant associations between any of the four SNPs and *change* in SBP or DBP during follow-up among boys (data not shown).

**Table 3:** Association between minor ACE2 alleles and blood pressure differences among males (NDIT Study, 1999–2005)

	**SBP, mmHg**			**DBP, mmHg**		
	**Beta (Confidence interval)**^**1**^			**Beta (Confidence interval)**^**1**^		
**SNP**^**2**^	**French Canadian**	**European**	**Other**	**French Canadian**	**European**	**Other**
rs2074192	−2.65 (−7.3, 2.0)	−3.32 (−6.1, -0.6)^3^	1.57 (−3.5, 6.6)	0.41 (−2.0, 2.8)	−2.32 (−3.8, -0.8)^4^	−1.18 (−4.1, 1.7)
rs233575	−1.05 (−5.3, 3.2)	3.59 (0.8, 6.4)^5^	−1.05 (−5.9, 3.8)	−2.88 (−5.0. -0.8)^6^	2.42 (0.8, 4.0)^7^	−1.24 (−4.0, 1.6)
rs2158083	−1.85 (−6.0, 2.3)	3.40 (0.6-6.2)^8^	−0.32 (−5.2, 4.6)	−2.66 (−4.8. -0.6)^9^	1.33 (−0.3-2.9)	−0.20 (−3.0, 2.6)
rs1978124	−2.01 (−6.0, 2.0)	2.00 (−0.8, 4.8)	1.79 (−3.0, 6.6)	−1.29 (−3.3, 0.8)	0.73 (−0.9, 2.4)	0.90 (−1.9, 3.7)

^1^Adjusted for height, and whether or not the participant was overweight or obese; ^2^Reference groups were the major genotypes in accordance with dbSNP database: G for rs2074192 and rs1978124; T for rs233575 and rs2158083; ^3^*p*-value=0.02; ^4^*p*-value=0.003; ^5^*p*-value=0.01; ^6^*p*-value=0.001; ^7^*p*-value=0.003; ^8^*p*-value=0.02; ^9^*p*-value=0.01.

### SNPs and blood pressure, or SNPS and blood pressure *change* (females)

In general none of the SNPs were associated with either SBP or DBP in females after adjustment for covariates (data not shown). However, there were statistically significant associations between several SNPs and *change* in SBP among females (Table 4). Females of European descent carrying the A allele of rs2074192 had a change in SBP during follow-up that was 1.5 mmHg lower than females of European descent carrying the G allele (*p* = 0.002). Females of European descent carrying the C allele of rs233575 and rs2158083 had a change in their SBP that was 1.9 mmHg (*p* = 0.002) and 1.20 mmHg (*p* = 0.04) higher, respectively, compared to females of European descent carrying the T allele.

**Table 4:** **Association between minor ACE2 alleles and blood pressure *change *** **among females (NDIT Study, 1999–2005)**

	**SBP, mmHg change**			**DBP, mmHg change**		
	**Beta (Confidence interval)**^**1**^			**Beta (Confidence interval)**^**1**^		
**SNP**^**2**^	**French Canadian**	**European**	**Other**	**French Canadian**	**European**	**Other**
rs2074192	−0.59 (−1.8, 0.6)	−1.54 (−0.6, -2.5)^3^	−1.48 (−3.2, 0.2)	−0.44 (−1.5, 0.6)	−0.20 (−1.0, 0.6)	−0.32 (−1.7, 1.0)
rs233575	0.30 (−1.3, 1.9)	1.95 (0.8, 3.1)^4^	1.41 (−0.6, 3.4)	0.87 (−0.3, 2.0)	0.67 (−0.1, 1.4)	−0.35 (−2.0, 1.3)
rs2158083	−0.05 (−1.4, 1.3)	1.20 (0.1, 2.3)^5^	2.83 (−0.2, 5.9)	0.39 (−0.7, 1.5)	0.53 (−0.2, 1.2)	0.06 (−2.3, 2.4)
rs1978124	0.34 (−1.0, 1.7)	0.78 (−0.3, 1.9)	0.04 (−1.4, 1.5)	−0.25 (−1.3, 0.8)	0.62 (−0.01, 1.2)	−0.45 (−1.6. 0.7)

^1^Adjusted for height, and whether or not the participant was overweight or obese; ^2^Reference groups were the major genotypes in accordance with dbSNP database: G for rs2074192 and rs1978124; T for rs233575 and rs2158083; ^3^*p*-value=0.002; ^4^*p*-value=0.002; ^5^*p*-value=0.04.

## Discussion

As a part of the renin-angiotensin aldosterone system, an important regulator of blood pressure homeostasis, ACE2 is believed to have a role in blood pressure regulation [9]. Indeed, ACE2 catalyzes the conversions of Angiotensin I and II into Angiotensin 1–7 which is known to have a vasodilator function [7,8]. Moreover, strong biological evidence from animal models suggests that the ACE2 gene is a regulator of blood pressure [7-12]. However, the human genetic literature is mixed with some studies reporting a relationship between ACE2 SNPs and blood pressure [15,16] while others do not [11,13,14].

To the best of our knowledge, our study is the first to investigate the association between tag SNPs in the ACE2 gene with blood pressure and blood pressure change in adolescents using multiple measures of blood pressure over five years of follow-up. Interestingly, we observed two associations (rs2074192 and rs233575) in males and females that were entirely driven by participants of European ancestry. Our results indicate that the ACE2 gene and more specifically, the major allele of rs2074192 and the minor allele of rs233575 significantly increase SBP and DBP among males of European ancestry, and significantly increase SBP change among females of European ancestry.

All previous studies on the ACE2 gene were carried out in adults using cross-sectional or case–control designs with single-point estimates, or follow-up time less than a month. In contrast, our study utilized nearly five years of follow-up among adolescents. The use of multiple blood pressure measurements in longitudinal data are less affected by random sources of variation than measurements taken at one time [27] and thus can provide stronger evidence for an association. Consistent with an intervention study among Han Chinese at high-risk for hypertension [28], rs2074192 was statistically significantly associated with DBP and SBP among the males in our study. Consistent with previous studies in German and Han Chinese populations, our results did not detect any associations between blood pressure and rs1978124 [11,14].

Our study is not without limitations. Candidate gene studies and Genome Wide Association Studies have both been criticized for their low replication of results, and candidate gene studies are additionally criticized for the subjectivity of their gene selection [29]. However, we selected the ACE2 gene based on animal models as well as the role of the gene in blood pressure homeostasis, and our results are consistent with previous studies conducted among adults. While candidate gene studies cannot provide an expansive analysis of all possible causative genes, they can provide insight into our understanding of biological pathways [30]. However, our study was limited to tag-SNPs of the ACE2 gene and future candidate gene studies would benefit from replication in larger studies. Due to restricting our analyses to homozygous females and stratifying by sex and ethnicity, our sample size varied between analyses and may be underpowered. Nevertheless, although we did not adjust for multiple testing, we found significant associations between tag SNPs and systolic blood pressure change among females that were consistent with the systolic blood pressure differences among males.

We acknowledge that restricting the analysis to homozygous females discards some information and can lead to a loss of power. Clayton (2008, 2009) has suggested tests of associations for SNPs located on the X chromosome that take into account X inactivation by giving a weight of ½ to the heterozygous genotype in females, essentially using an additive coding for the genotype (i.e., genotypes are coded as 0, 1, or 2 for the number of minor alleles) [31,32]. We analyzed our data using this model in females and obtained very similar results as those obtained by comparing the two homozygous genotypes (Additional file 3: Table S3 and Additional file 4: Table S4). We also used other models of X inactivation, corresponding to dominant (Additional file 5: Table S5 and Additional file 6: Table S6) and recessive coding of the genotypes (Additional file 7: Table S7 and Additional file 8: Table S8) [33], and obtained similar results. Given the uncertainty in the model of X inactivation, we feel that it is more conservative to present our analyses excluding heterozygous females.

While 92% of our participants were Canadian-born, 28% of their mothers and 37% of their fathers were foreign-born, and ethnicity has been shown to modify the association between SNPs and blood pressure [34]. Our tag SNPs were selected based on the HapMap data of northern and western European ancestry, which may not have provided sufficient coverage in other ethnicities. However, the “Other” ethnicity group was heterogeneous, and there were too few participants of any one ethnicity to warrant selecting tag SNPs. While insufficient coverage could contribute to the absence of association detected in the “Other” ethnicity group, significant results in this group would be extremely hard to interpret given the possibility of population stratification bias. Although most SNPs were in Hardy-Weinberg Equilibrium, there was evidence of a significant difference in the allele frequencies of rs2074192 between males and females of “French-Canadian” and “Other” descent. Lastly, puberty in females ends at approximately 15 years of age, and it is possible that blood pressure trajectories change at this time. However we were not able to assess this given that we only had three blood pressure measurements over time. Additional studies allowing for two different slopes in females (before and after age 15 years) may provide additional insight.

## Conclusion

Despite these limitations, our results suggest that several ACE2 gene SNPs are associated with either blood pressure levels or blood pressure change among males and females of European ancestry. Whether these SNPs could be new therapeutic targets, or potential primordial targets in persons of European descent should be further explored.

## Abbreviations

ACE2: Angiotensin-converting enzyme-2; BMI: Body mass index; DBP: Diastolic blood pressure; HTN: Hypertension; NDIT: Nicotine dependence in teens study; SBP: Systolic blood pressure; SNPs: Single nucleotide polymorphisms.

## Competing interests

The authors have no competing interests to declare.

## Author’s contributions

LM performed the statistical analysis, interpreted the data, and drafted the manuscript. LK contributed to the interpretation of the data and participated in the drafting of the manuscript. JOL conceived of and designed the study, obtained the funding, participated in its coordination, contributed to the interpretation of the data, and participated in the drafting of the manuscript. LP, PH, JT, conceived of and designed the study, obtained the funding, contributed to the interpretation of the data, and participated in the drafting of the manuscript. GP participated in the conception of and design of the study, contributed to the interpretation of the data, and participated in the drafting of the manuscript. M-H R-G and AL contributed to the statistical analysis, interpretation of the data and participated in the drafting of the manuscript. All authors read and approved the final manuscript.

## Pre-publication history

The pre-publication history for this paper can be accessed here:

http://www.biomedcentral.com/1471-2350/14/117/prepub

## Supplementary Material

Additional file 1: Table S1 Linkage disequilibrium between SNPs in homozygous females (NDIT Study, 1999–2005). Click here for file

Additional file 2: Table S2 Mean blood pressure (mmHg) at baseline according to genotype among homozygous participants (NDIT Study, 1999–2005). Click here for file

Additional file 3: Table S3 Association between minor ACE2 alleles and blood pressure differences among females (NDIT Study, 1999–2005) using the additive model. Click here for file

Additional file 4: Table S4 Association between minor ACE2 alleles and blood pressure change among females (NDIT Study, 1999–2005) using the additive model. Click here for file

Additional file 5: Table S5 Association between minor ACE2 alleles and blood pressure differences among females (NDIT Study, 1999–2005) using the dominant model. Click here for file

Additional file 6: Table S6 Association between minor ACE2 alleles and blood pressure change among females (NDIT Study, 1999–2005) using the dominant model. Click here for file

Additional file 7: Table S7 Association between minor ACE2 alleles and blood pressure differences among females (NDIT Study, 1999–2005) using the recessive model. Click here for file

Additional file 8: Table S8 Association between minor ACE2 alleles and blood pressure change among females (NDIT Study, 1999–2005) using the recessive model. Click here for file
