# Supplementary material for: The association between the angiotensin-converting enzyme-2 gene and blood pressure in a cohort study of adolescents
Source: BMC Med Genet. 2013 Nov 5;14:117. doi: 10.1186/1471-2350-14-117 (PMC4228362; doi:10.1186/1471-2350-14-117)
Supplement: Additional file 1: Table S1 — Linkage disequilibrium between SNPs in homozygous females (NDIT Study, 1999–2005). [file 1471-2350-14-117-S1.doc]

**Supplementary Table A Linkage disequilibrium between SNPs in homozygous females** **NDIT Study, 1999-2005**

|  |  | SNP^1,2^ | | | |
| --- | --- | --- | --- | --- | --- |
|  |  | rs2074192 | rs233575 | rs2158083 | rs1978124 |
| **SNP** | rs2074192 |  | -0.35 | -0.21 | -0.03 |
|  | rs233575 |  |  | 0.59 | 0.25 |
|  | rs2158083 |  |  |  | 0.41 |
|  | ^1^R^2^; ^2^all *p*-values <0.05 | | | | |
