# Supplementary material for: The association between the angiotensin-converting enzyme-2 gene and blood pressure in a cohort study of adolescents
Source: BMC Med Genet. 2013 Nov 5;14:117. doi: 10.1186/1471-2350-14-117 (PMC4228362; doi:10.1186/1471-2350-14-117)
Supplement: Additional file 2: Table S2 — Mean blood pressure (mmHg) at baseline according to genotype among homozygous participants (NDIT Study, 1999–2005). [file 1471-2350-14-117-S2.doc]

**Supplementary Table B Mean blood pressure (mmHg) at baseline according to genotype among homozygous participants** (**NDIT Study, 1999-2005)**

|  |  | **Male^1,2^** | | | | | |  | **Female^1,2^** | | | | | |
| --- | --- | --- | --- | --- | --- | --- | --- | --- | --- | --- | --- | --- | --- | --- |
|  |  | **French Canadian** | | **European** | | **Other** | |  | **French Canadian** | | **European** | | **Other** | |
|  | **SNP (Minor/Major)^3^** | **Minor** | **Major** | **Minor** | **Major** | **Minor** | **Major** |  | **Minor** | **Major** | **Minor** | **Major** | **Minor** | **Major** |
| SBP | rs2074192 (A/G) | 105 | 107 | 104 | 106 | 105 | 103 |  | 106 | 106 | 105 | 102 | 111 | 105 |
|  | rs233575 (C/T) | 106 | 108 | 108 | 104* | 105 | 104 |  | 105 | 107 | 101 | 106 | 111 | 104 |
|  | rs2158083 (C/T) | 105 | 108 | 107 | 104 | 105 | 103 |  | 104 | 107 | 104 | 105 | 114 | 103 |
|  | rs1978124 (A/G) | 105 | 108 | 105 | 104 | 106 | 103 |  | 104 | 105 | 102 | 104 | 107 | 103 |
|  |  |  |  |  |  |  |  |  |  |  |  |  |  |  |
| DBP | rs2074192 (A/G) | 57 | 57 | 53 | 56* | 55 | 56 |  | 59 | 57 | 55 | 55 | 57 | 57 |
|  | rs233575 (C/T) | 56 | 57 | 56 | 54* | 55 | 56 |  | 55 | 59* | 55 | 56 | 59 | 56 |
|  | rs2158083 (C/T) | 56 | 57 | 55 | 54 | 56 | 56 |  | 55 | 59* | 56 | 56 | 58 | 56 |
|  | rs1978124 (A/G) | 56 | 57 | 55 | 55 | 56 | 55 |  | 58 | 59 | 55 | 56 | 56 | 55 |
| ^1^Depending on the SNP, n ranged from 142 to 150 female, and 257 to 264 male participants; ^2^* p-value <0.05 between major and minor genotypes; ^3^Categorizations of major and minor genotypes in accordance with dbSNP database | | | | | | | | | | | | | | |
