# Supplementary material for: The association between the angiotensin-converting enzyme-2 gene and blood pressure in a cohort study of adolescents
Source: BMC Med Genet. 2013 Nov 5;14:117. doi: 10.1186/1471-2350-14-117 (PMC4228362; doi:10.1186/1471-2350-14-117)
Supplement: Additional file 3: Table S3 — Association between minor ACE2 alleles and blood pressure differences among females (NDIT Study, 1999–2005) using the additive model. [file 1471-2350-14-117-S3.doc]

**Supplementary Table C Association between minor ACE2 alleles and blood pressure differences among females (NDIT Study, 1999-2005) using the additive model**

|  | **SBP, mmHg** | | |  | **DBP, mmHg** | | |
| --- | --- | --- | --- | --- | --- | --- | --- |
|  | **Beta (Confidence Interval)^1,2^** | | |  | **Beta (Confidence Interval)^1,2^** | | |
| **SNP^3^** | **French  Canadian** | **European** | **Other** |  | **French  Canadian** | **European** | **Other** |
| rs2074192 | 0.34 (-2.3, 3.0) | -0.43 (-2.1, 1.2) | 0.39 (-2.4, 3.2) |  | 0.76 (-0.7, 2.2) | -0.66 (-1.8, 0.5) | -0.18 (-1.9, 1.6) |
| rs233575 | -0.78 (-3.6, 2.1) | -0.11 (-2.0, 1.7) | 1.79 (-1.4, 5.0) |  | -0.84 (-2.4, -0.7) | 0.39 (-0.9, 1.7) | -0.19 (-2.1, 1.8) |
| rs2158083 | -1.33 (-4.1, 1.4) | 0.96 (-0.8,2.8) | 1.22 (-2.3, 4.7) |  | -0.6 (-2.2, 0.9) | 0.74 (-0.5, 2.0) | -0.14 (-2.3, 2.0) |
| rs1978124 | -1.00 (-3.7, 1.7) | -0.04 (-1.7, 1.6) | 1.59 (-1.2, 4.3) |  | -0.50 (-2.0, 1.0) | 0.34 (-0.8, 1.5) | 0.51 (-1.2, 2.2) |
| ^1^Adjusted for height, and whether or not the participant was overweight or obese; ^2^* p<0.05, ** p<0.01; ^3^Genotype coded as 0, 1, or 2 for the number of minor alleles in accordance with dbSNP database: G for rs2074192 and rs1978124; T for rs233575 and rs2158083 | | | | | | | |
