# Supplementary material for: The association between the angiotensin-converting enzyme-2 gene and blood pressure in a cohort study of adolescents
Source: BMC Med Genet. 2013 Nov 5;14:117. doi: 10.1186/1471-2350-14-117 (PMC4228362; doi:10.1186/1471-2350-14-117)
Supplement: Additional file 4: Table S4 — Association between minor ACE2 alleles and blood pressure change among females (NDIT Study, 1999–2005) using the additive model. [file 1471-2350-14-117-S4.doc]

**Supplementary Table D Association between minor ACE2 alleles and blood pressure change among females (NDIT Study, 1999-2005) using the additive model**

|  | **SBP, mmHg change** | | |  | **DBP, mmHg change** | | |
| --- | --- | --- | --- | --- | --- | --- | --- |
|  | **Beta (Confidence Interval)^1^** | | |  | **Beta (Confidence Interval)^1^** | | |
| **SNP^2^** | **French  Canadian** | **European** | **Other** |  | **French  Canadian** | **European** | **Other** |
| rs2074192 | -0.15 (-0.8, 0.5) | -0.87 (-1.4, -0.4)^3^ | -0.59 (-1.3, 0.1) |  | -0.16 (-0.6, 0.3) | -0.11 (-0.5, 0.2) | -0.13 (-0.7, 0.4) |
| rs233575 | 0.16 (-0.6, 0.9) | 0.96 (0.4, 1.5)^4^ | 0.26 (-0.5, 1.1) |  | 0.38 (-0.1, 0.9) | 0.27 (-0.1, 0.6) | -0.26 (-0.9 0.4) |
| rs2158083 | 0.09 (-0.6, 0.8) | 0.68 (0.1, 1.2)^5^ | 0.01 (-0.9, 0.9) |  | 0.23 (-0.3, 0.8) | 0.18 (-0.2, 0.5) | -0.44 (-1.1, 0.2) |
| rs1978124 | 0.16 (-0.5, 0.9) | 0.34 (-0.2, 0.9) | -0.15 (-0.8, 0.5) |  | -0.08 (-0.6, 0.4) | 0.33 (-0.01, 0.7) | -0.42 (-1.0, 0.1) |
| ^1^Adjusted for height, and whether or not the participant was overweight or obese; ^2^Genotype coded as 0, 1, or 2 for the number of minor alleles in accordance with dbSNP database: G for rs2074192 and rs1978124; T for rs233575 and rs2158083; ^3^*p*-value=0.001; ^4^*p*-value=0.0007; ^5^*p*-value=0.01 | | | | | | | |
