# Supplementary material for: The association between the angiotensin-converting enzyme-2 gene and blood pressure in a cohort study of adolescents
Source: BMC Med Genet. 2013 Nov 5;14:117. doi: 10.1186/1471-2350-14-117 (PMC4228362; doi:10.1186/1471-2350-14-117)
Supplement: Additional file 5: Table S5 — Association between minor ACE2 alleles and blood pressure differences among females (NDIT Study, 1999–2005) using the dominant model. [file 1471-2350-14-117-S5.doc]

**Supplementary Table E Association between minor ACE2 alleles and blood pressure differences among females (NDIT Study, 1999-2005) using the dominant model**

|  | **SBP, mmHg** | | |  | **DBP, mmHg** | | |
| --- | --- | --- | --- | --- | --- | --- | --- |
|  | **Beta (Confidence Interval)^1,2^** | | |  | **Beta (Confidence Interval)^1,2^** | | |
| **SNP^3^** | **French  Canadian** | **European** | **Other** |  | **French  Canadian** | **European** | **Other** |
| rs2074192 | 2.66 (-1.3, 6.6) | -1.39 (-4.0, 1.2) | -1.41 (-5.4, 2.6) |  | 1.80 (-0.4, 4.0) | -1.26 (-3.1, 0.6) | -1.03 (-3.5, 1.4) |
| rs233575 | -0.05 (-3.9, 3.8) | -0.83 (-3.4, 1.7) | 0.70 (-3.2, 4.6) |  | -0.74 (-2.9, 1.4) | -0.13 (-1.9, 1.7) | -0.97 (-3.3, 1.4) |
| rs2158083 | -0.59 (-4.4, 3.2) | -0.08 (-2.5, 2.4) | 0.66 (-3.3, 4.6) |  | 0.07 (-2.1, 2.2) | 0.31 (-1.4, 2.1) | -0.28 (-2.7, 2.1) |
| rs1978124 | 1.30 (-3.0, 5.6) | 1.32 (-1.4, 4.1) | 2.29 (-1.6, 6.1) |  | -0.07 (-2.5, 2.4) | 0.65 (-1.3, 2.6) | 0.98 (-1.4, 3.3) |
| ^1^Adjusted for height, and whether or not the participant was overweight or obese; ^2^* p<0.05, ** p<0.01; ^3^Reference groups were the homozygote major genotypes in accordance with dbSNP database: G for rs2074192 and rs1978124; T for rs233575 and rs2158083 | | | | | | | |
