# Supplementary material for: The association between the angiotensin-converting enzyme-2 gene and blood pressure in a cohort study of adolescents
Source: BMC Med Genet. 2013 Nov 5;14:117. doi: 10.1186/1471-2350-14-117 (PMC4228362; doi:10.1186/1471-2350-14-117)
Supplement: Additional file 6: Table S6 — Association between minor ACE2 alleles and blood pressure change among females (NDIT Study, 1999–2005) using the dominant model. [file 1471-2350-14-117-S6.doc]

**Supplementary Table F Association between minor ACE2 alleles and blood pressure change among females (NDIT Study, 1999-2005) using the dominant model**

|  | **SBP, mmHg** | | |  | **DBP, mmHg** | | |
| --- | --- | --- | --- | --- | --- | --- | --- |
|  | **Beta (Confidence Interval)^1^** | | |  | **Beta (Confidence Interval)^1^** | | |
| **SNP^2^** | **French  Canadian** | **European** | **Other** |  | **French  Canadian** | **European** | **Other** |
| rs2074192 | 0.57 (-0.4, 1.6) | -1.64 (-2.4, -0.9)^3^ | -0.31 (-1.3, 0.7) |  | 0.08 (-0.6, 0.8) | -0.40 (-0.9, 0.1) | -0.07 (-0.9, 0.7) |
| rs233575 | 0.17 (-0.8, 1.1) | 1.05 (0.3, 1.8)^4^ | 0.11 (-0.9, 1.1) |  | 0.41 (-0.3, 1.1) | 0.15 (-0.4, 0.7) | -0.38 (-1.1, 0.4) |
| rs2158083 | 0.28 (-0.7, 1.2) | 0.98 (0.2, 1.7)^5^ | -0.19 (-1.2, 0.8) |  | 0.35 (-0.4, 1.1) | -0.01 (-0.5, 0.5) | -0.57 (-1.3, 0.2) |
| rs1978124 | 0.10 (-1.0, 1.2) | 0.77 (-0.1, 1.6) | -0.39 (-1.3, 0.5) |  | 0.34 (-0.5, 1.2) | 0.36 (-0.2, 0.9) | -0.80 (-1.5, -0.1)^6^ |
| ^1^Adjusted for height, and whether or not the participant was overweight or obese; ^2^Reference groups were the homozygote major genotypes in accordance with dbSNP database: G for rs2074192 and rs1978124; T for rs233575 and rs2158083; ^3^p=<.0001; ^4^p=0.007; ^5^p=0.01; ^6^p=0.03 | | | | | | | |
