# Supplementary material for: The association between the angiotensin-converting enzyme-2 gene and blood pressure in a cohort study of adolescents
Source: BMC Med Genet. 2013 Nov 5;14:117. doi: 10.1186/1471-2350-14-117 (PMC4228362; doi:10.1186/1471-2350-14-117)
Supplement: Additional file 7: Table S7 — Association between minor ACE2 alleles and blood pressure differences among females (NDIT Study, 1999–2005) using the recessive model. [file 1471-2350-14-117-S7.doc]

**Supplementary Table G Association between minor ACE2 alleles and blood pressure differences among females (NDIT Study, 1999-2005) using the recessive model**

|  | **SBP, mmHg** | | |  | **DBP, mmHg** | | |
| --- | --- | --- | --- | --- | --- | --- | --- |
|  | **Beta (Confidence Interval)^1^** | | |  | **Beta (Confidence Interval)^1^** | | |
| **SNP^2^** | **French  Canadian** | **European** | **Other** |  | **French  Canadian** | **European** | **Other** |
| rs2074192 | -2.71 (-7.3, 1.9) | 0.34 (-2.5, 3.2) | 4.30 (-0.1, 9.6) |  | -0.1 (-2.7, 2.5) | -0.40 (-2.4, 1.6) | 1.21 (-2.1, 4.5) |
| rs233575 | -3.15 (-8.9, 2.6) | 1.20 (-2.3, 4.7) | 9.58 (1.5, 17.7)^3^ |  | -1.80 (-5.0, 1.4) | 1.66 (-0.8, 4.1) | 3.12 (-1.9, 8.2) |
| rs2158083 | -4.0 (-9.5, 1.4) | 3.36 (0.2, 6.5)^4^ | 8.03 (-3.5, 19.6) |  | -2.53 (-5.6, 0.5) | 2.03 (-0.2, 4.3) | 0.88 (-6.2, 8.0) |
| rs1978124 | -4.0 (-8.3, 0.4) | -1.25 (-3.9, 1.4) | 1.93 (-4.0, 7.8) |  | -1.25 (-3.7, 1.2) | 0.27 (-1.6, 2.1) | 0.09 (-3.5, 3.7) |
| ^1^Adjusted for height, and whether or not the participant was overweight or obese; ^2^Reference groups were the homozygote major and heterozygote genotypes in accordance with dbSNP database: G for rs2074192 and rs1978124; T for rs233575 and rs2158083; ^3^p=0.02; ^4^p=0.04; | | | | | | | |
