# Supplementary material for: The association between the angiotensin-converting enzyme-2 gene and blood pressure in a cohort study of adolescents
Source: BMC Med Genet. 2013 Nov 5;14:117. doi: 10.1186/1471-2350-14-117 (PMC4228362; doi:10.1186/1471-2350-14-117)
Supplement: Additional file 8: Table S8 — Association between minor ACE2 alleles and blood pressure change among females (NDIT Study, 1999–2005) using the recessive model. [file 1471-2350-14-117-S8.doc]

**Supplementary Table H Association between minor ACE2 alleles and blood pressure change among females (NDIT Study, 1999-2005) using the recessive model**

|  | **SBP, mmHg change** | | |  | **DBP, mmHg change** | | |
| --- | --- | --- | --- | --- | --- | --- | --- |
|  | **Beta (Confidence Interval)^1^** | | |  | **Beta (Confidence Interval)^1^** | | |
| **SNP^2^** | **French  Canadian** | **European** | **Other** |  | **French  Canadian** | **European** | **Other** |
| rs2074192 | -1.29 (-2.4, -0.1)^3^ | -0.53 (-1.4, 0.4) | -1.54 (-2.8, -0.3)^4^ |  | -0.63 (-1.5, 0.2) | 0.15 (-0.4, 0.8) | -0.29 (-1.3, 0.7) |
| rs233575 | 0.29 (-1.2, 1.8) | 1.50 (0.4, 2.6)^5^ | -1.29 (-0.8, 3.4) |  | 0.68 (-0.4, 1.8) | 0.70 (-0.03, 1.4) | -0.14 (-1.8, 1.5) |
| rs2158083 | -0.22 (-1.6, 1.2) | 0.70 (-0.3, 1.7) | 2.14 (-0.9, 5.2) |  | 0.19 (-0.8, 1.2) | 0.64 (-0.04, 1.3) | 0.27 (-2.1, 2.6) |
| rs1978124 | 0.32 (-0.8, 1.4) | 0.17 (-0.6, 1.0) | 0.26 (-1.2, 1.7) |  | -0.57 (-1.4, 0.3) | 0.50 (-0.04, 1.0) | -0.03 (-1.2, 1.1) |
| ^1^Adjusted for height, and whether or not the participant was overweight or obese; ^2^Reference groups were the homozygote major and heterozygote genotypes in accordance with dbSNP database: G for rs2074192 and rs1978124; T for rs233575 and rs2158083; ^3^*p*-value=0.03; ^4^*p*-value=0.02; ^5^*p*-value=0.001; | | | | | | | |
